# Maternal Mortality in Pakistan: Demographic, Temporal, and Contextual Insights From the Three Delays Model

**DOI:** 10.7759/cureus.98424

**Published:** 2025-12-03

**Authors:** Noor Ullah Khan, Nida Asif, Muhammad A Miraj, Hamza Khalid, Talha Awan, Maaz Ahmed Khan, Nimra Khalid, Phyu Thet Mon, Alyshba Shafi

**Affiliations:** 1 Public Health, Health Services Academy, Islamabad, PAK; 2 Pediatric Medicine, Mardan Medical Complex, Mardan, PAK; 3 Internal Medicine, Pakistan Institute of Medical Sciences, Islamabad, PAK; 4 Otolaryngology-Head and Neck Surgery, Pakistan Institute of Medical Sciences, Islamabad, PAK; 5 Family Medicine, One Health Medical Services, Islamabad, PAK; 6 Physiology, Shifa Tameer-E-Millat University, Islamabad, PAK; 7 Internal Medicine, Bahosi Hospital, Yangon, MMR; 8 Medicine, Peshawar Medical College, Peshawar, PAK

**Keywords:** demographic and health survey, maternal health services, maternal mortality, pakistan maternal deaths, three delays model

## Abstract

Background

Maternal mortality remains a pressing health concern, especially in low‑ and middle‑income countries. Understanding the demographic, temporal, and contextual factors that lead to these deaths is essential for designing effective interventions. This study aimed to examine maternal mortality through the lens of the three delays model using data from Pakistan.

Methods

We conducted a retrospective analysis of the Pakistan Maternal Mortality Survey 2018‑2019, drawing on the Pakistan Demographic and Health Survey Verbal Autopsy dataset. Descriptive statistics and stratified analyses were used to profile maternal deaths by demographic characteristics, timing, and place of death. We also quantified delays in deciding to seek care, reaching a facility, and receiving treatment.

Results

Of the 1,177 maternal deaths analyzed, the mean age was 34 years. Most deaths occurred in health facilities and were classified as direct obstetric causes such as hemorrhage and sepsis. Women experienced an average delay of 3.8 days in deciding to seek care, 3.7 hours in reaching a facility, and 7.6 minutes in receiving treatment. More than half of the deaths occurred within 42 days postpartum. Financial hardship, geographic isolation, and limited resources emerged as prominent reasons for delay. Women who reached a health facility were less likely to die on the first day of admission than those who did not.

Conclusions

Maternal mortality in Pakistan reflects a web of sociodemographic inequalities and systemic shortcomings. Addressing these deaths requires more than clinical solutions. It calls for policies that improve the timeliness and quality of maternal health services, tackle financial and geographic barriers, and strengthen the healthcare system. Interventions grounded in the three delays framework could help reduce maternal mortality and advance maternal health equity in low‑resource settings.

## Introduction

Maternal mortality, the death of a woman during pregnancy, during childbirth, or within the postpartum period, remains one of the most persistent public health challenges worldwide. Despite advances in obstetric care and global initiatives to reduce maternal deaths, mortality rates remain unacceptably high in many low‑ and middle‑income countries (LMICs) [1]. These deaths are not solely medical events; they reflect the intersection of socioeconomic conditions, cultural practices, and health system performance [2]. Understanding the multifaceted drivers of maternal mortality is therefore essential for designing effective policies and interventions.

A number of frameworks have been developed to characterize why women die from pregnancy‑related causes. One of the most widely used is the three delays model proposed by Thaddeus and Maine [3]. It highlights three critical junctures where failures can occur: delay in deciding to seek care, delay in reaching a health facility, and delay in receiving appropriate care upon arrival [4]. Subsequent studies have employed this framework to demonstrate how sociocultural norms, financial constraints, geographic barriers, and health system deficiencies interact to impede timely care.

Pakistan is among the countries where maternal mortality ratios remain high relative to global targets [5]. While previous research has identified individual risk factors, there is a paucity of comprehensive analyses that simultaneously examine demographic characteristics, temporal patterns of death, and contextual reasons for delay. Moreover, few studies have used nationally representative data to assess how these factors intersect. Addressing this gap, we undertook a retrospective analysis of the Pakistan Maternal Mortality Survey (PMMS) 2018‑2019, a component of the Pakistan Demographic and Health Survey (PDHS) program [6]. By applying the three delays model to this dataset, we aimed to elucidate the demographic, temporal, and contextual determinants of maternal deaths in Pakistan. The goal of this research is to provide evidence that can inform targeted interventions and policies to reduce maternal mortality and advance maternal health equity.

## Materials and methods

Study design and data source

We conducted a retrospective analysis of secondary data from the PMMS 2018‑2019, which is part of the PDHS program. The PDHS is a nationally representative cross‑sectional survey carried out by the National Institute of Population Studies (NIPS) in collaboration with the Inner City Fund (ICF) International and sponsored by the United States Agency for International Development (USAID). We drew on the PDHS "Verbal Autopsy" dataset (PKVA7ASV), which collects demographic characteristics and detailed circumstances of death for women of reproductive age.

Sampling and study population

The PDHS uses a stratified two‑stage cluster sampling design. Pakistan is stratified by province and by urban or rural residence; clusters are selected with probability proportional to size, and households are then systematically sampled within clusters. Our study population included all maternal deaths recorded in the PMMS dataset. For the purposes of this study, we defined a maternal death as the death of a woman during pregnancy, during childbirth, or within one year postpartum, irrespective of cause, consistent with PDHS definitions. Cases lacking a recorded place of treatment were excluded.

Data preparation

We reviewed the dataset for completeness and consistency. Variable formats were standardized, and duplicate entries were removed. Interviews containing missing data for more than 30% of variables were excluded from analysis to ensure data reliability. For the remaining interviews, missing numerical values were handled using median imputation. Specifically, respondents were stratified by district, and missing values were replaced with the median calculated from all respondents within the corresponding district. Sample sizes vary across analyses because not all variables were available for each case. Each table and figure is based on the subset of deaths with complete data for the variables under study.

Statistical analysis

A number of different analyses were undertaken based on available data. Descriptive statistics summarized demographic characteristics and maternal health indicators. Stratified analyses examined the association between demographic factors and the place of death (health facility versus home or in transit). A two‑by‑two contingency table was constructed to estimate the relative risk (RR) of dying on the first day after reaching a health facility. To explore temporal aspects of care‑seeking, we applied the three delays framework, calculating mean delays in deciding to seek care, reaching a facility, and receiving treatment. Because the Verbal Autopsy dataset does not record the exact interval between arrival at a facility and initiation of treatment, we were unable to quantify the third delay. Therefore, we have used the reported reasons for referral to another facility as an indirect measure to understand the delays in receiving appropriate care indicating a longer third delay. All analyses were performed using IBM SPSS Statistics for Windows, V. 25.0 (IBM Corp., Armonk, NY, USA).

Ethical considerations

Ethical approval for the PDHS was obtained from the Research Ethics Committee of the National Bioethics Committee of Pakistan (approval number: 4‑87/NBC‑346/19). Because we analyzed anonymized secondary data, no additional ethical approval was required. This article was previously posted to the Research Square preprint server on May 7, 2024 [7].

## Results

A total of 1,177 maternal deaths were included, with a mean age of 34 years (SD ±10.2). Most women had completed at least primary education (707, 60.1%), though fewer progressed to secondary or higher levels. Care-seeking before death was common (1,029, 87.4%), and government hospitals were the usual first point of contact (670, 65.1%), followed by private facilities (280, 27.2%) and, rarely, home-based or traditional care. General practitioners provided most of the initial management (896, 87.1%), with specialist involvement remaining limited. The vast majority of deaths occurred within health facilities (961, 93.4%), with relatively few at home or in transit (Table 1).

**Table 1:** Demographic characteristics of maternal deaths (n=1,177) LHV: lady health visitor

Demographic characteristics	Frequency (n)	Percentage (%)
Mean age of death (years)	34.12	-
Educational status		
None	29	2.5
Primary (1-8)	707	60.1
Secondary (9-10)	236	20.1
Higher secondary and above	205	17.4
Treatment sought before death		
Yes	1,029	87.4
No	148	12.6
Place of death (n=1,029)		
Home or in transit	68	6.6
Health facility	961	93.4
First place of treatment (n=1,029)		
Home	68	6.6
Government hospital	670	65.1
Rural health center/basic health unit/government facility	11	1.1
Private hospital/clinic	280	27.2
Healthcare providers at the place of treatment (n=1,029)		
Hakim	1	0.1
Spiritual healer	2	0.2
Homeopath	3	0.3
Dispenser	21	2
Nurse/midwife/LHV	17	1.7
General doctor	896	87.1
Obstetrician/specialist	89	8.6

**Figure 1:**
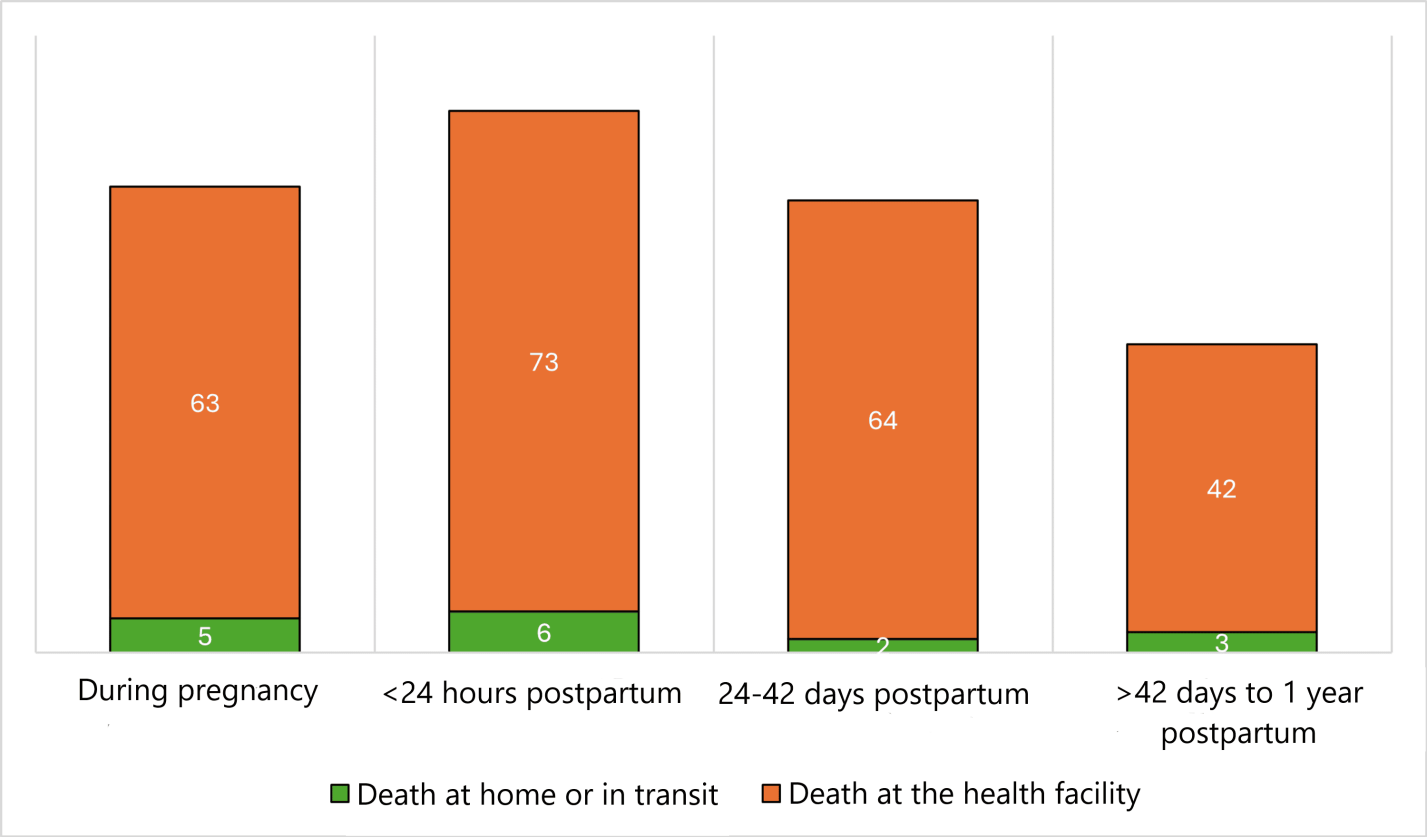
Distribution of maternal deaths by time period and location (n=258)

Maternal deaths occurred across all stages of pregnancy, childbirth, and the postpartum period, with over half taking place between delivery and 42 days postpartum (147, 57%). Across every time point, deaths were overwhelmingly facility-based, with the large majority occurring in health facilities (242, 93.8%) rather than at home or in transit (16, 6.2%) (Figure 1).

To assess the early impact of facility-based care, we compared first-day mortality between women who reached a health facility and those who died at home or in transit. First-day deaths were less common among women who accessed a facility, with an RR of 0.78, indicating a modest protective effect during the initial 24 hours of care (Table 2).

**Table 2:** Contingency table of deaths versus survival on the first day at health facility (n=258) The relative risk (RR) calculated from the 2×2 table is as follows: RR=a/(a+b)÷c/(c+d)​ RR=73/(73+165)÷8/(8+12) RR=0.305/0.3889 RR=0.78

	Death on the first day	Alive on the first day	Total
Reached	73 (30.7%)	165 (69.3%)	238 (100%)
Not reached	8 (40%)	12 (60%)	20 (100%)

Analysis of the three delays showed substantial waiting times at each stage. Decisions to seek care took the longest, averaging several days, followed by delays in reaching a facility and shorter waits to receive treatment. These delays were consistently longer among women who died in health facilities. For example, the decision to seek care interval was notably prolonged for facility deaths (3.8 days vs. 1.1 days at home), and the additional time required to obtain appropriate care after referral was also greater (13.5 minutes vs. negligible at home) (Table 3).

**Table 3:** Average intervals for each of the three delays *For deaths at home category, type 2 delay signifies the time it took for healthcare providers (doctors, LHVs, CAM providers, etc.) to reach the patient's location, while for deaths at health facilities category, it signifies the time it took to reach the facility. LHVs: lady health visitors; CAM: complementary and alternative medicine

Delay type	Deaths at home	Deaths at health facilities
Type 1 delay (delay in decision to seek care)	1.1 days per case	3.8 days per case
Type 2 delay* (delay in accessing care)	1.8 hours per case	3.8 hours per case
Type 3 delay (delay in receiving appropriate care)	0 minutes per case	13.5 minutes per case

Reasons for delays differed across the three stages. For the first delay, over one-third of women died before any decision to seek care was made (381, 32.4%), with many others not recognizing the severity of their condition or facing geographic and transport barriers. Financial constraints were less commonly cited. During the second delay, distance to the facility was the predominant obstacle (438, 37.2%), followed by limited awareness of illness severity and lack of funds. For the third delay, referrals frequently reflected gaps in facility readiness with about half of the women transferred in search of better care (587, 49.9%), and another quarter because essential resources or equipment were unavailable (256, 21.7%), with smaller numbers referred for specific unmanaged clinical conditions (Table 4).

**Table 4:** Reported reasons contributing to the three delays in maternal care (n=1,177)

	Frequency (n)	Percentage (%)
Reasons for the first delay		
No decision made	381	32.4
Did not realize seriousness	260	22.1
No treatment necessary	162	13.8
Other	145	12.3
Transport issue	34	2.8
Too far	98	8.3
Cost issues	89	7.6
Good care at home	8	0.7
Reasons for the second delay		
Hospital too far	438	37.2
Did not realize seriousness	350	29.7
Lack of funds	200	17
No transport	63	5.4
Other	63	5.4
Nighttime	50	4.2
Husband away	13	1.1
Reasons for the third delay (reasons for referral)		
To get better care	587	49.9
No proper arrangements for resolving problems	256	21.7
No equipment for operation	109	9.3
Other	91	7.7
High blood pressure	75	6.4
No doctor available	37	3.1
No arrangements for transfusing blood	22	1.9

Direct obstetric complications were the leading causes of death, accounting for most cases with identified causes (150, 65.5%), particularly postpartum hemorrhage, sepsis, and hypertensive disorders. Indirect causes, including largely pre-existing medical conditions exacerbated by pregnancy, were less common (20, 8.7%). Smaller proportions of deaths were classified as probable, coincidental, or late maternal deaths (Table 5).

**Table 5:** Causes of maternal deaths (n=229) The bold texts signify the category of death, while the normal font signifies the sub-categories of direct obstetric deaths.

Type and cause	Death at home or in transit (n)	Percentage (%)	Death at the facility	Percentage (%)	Total (n)
Direct obstetric death	36	24	114	76	150
Postpartum hemorrhage	9	26.5	25	73.5	34
Puerperal sepsis	-	0	10	100	10
Preeclampsia	3	13.6	19	86.4	22
Surgical complication	2	22.2	7	77.8	9
Eclampsia	4	57.1	3	42.9	7
Premature separation of the placenta	2	33.3	4	66.7	6
Other	16	25.8	47	74.2	63
Indirect obstetric death	3	15	17	85	20
Probable obstetric death	1	50	1	50	2
Coincidental obstetric death	10	47.6	11	52.4	21
Late maternal death	4	11.1	32	88.9	36

## Discussion

Our analysis of maternal deaths from the PMMS identified several patterns. The average age at death was 34 years, and most women died in health facilities, with more than half of deaths occurring within the early postpartum period. Direct obstetric causes, particularly postpartum hemorrhage, sepsis, and hypertensive disorders, accounted for most deaths. Socioeconomic factors, geographic isolation, and inadequate facility resources collectively contributed to these delays. Women experienced long delays in deciding to seek care and in reaching facilities; those who did reach care were less likely to die within the first 24 hours than women who died at home or en route. Although early survival differed between the two groups, the RR suggests that even minimal contact with the health system may offer some immediate stabilization that is not available at home. However, this early advantage does not appear to extend beyond the first day, as reflected in the high overall proportion of facility deaths, underscoring persistent gaps in sustained emergency and inpatient care.

These findings align with and extend existing literature. Globally, severe bleeding, infections, hypertensive disorders, and other obstetric complications are responsible for roughly 75% of maternal deaths [8], and most deaths from hemorrhage and sepsis occur after delivery [9]. The mean age at death in our study was 34 years. Evidence from multiple LMICs indicates that the risk of maternal mortality increases for women over 35. For example, an analysis of 571,321 pregnancies across six LMICs found that maternal age >35 was associated with a 43% higher risk of death [10]. Low educational attainment, nulliparity, and parity ≥2 were also significant risk factors in that cohort [10], highlighting the intersection of sociodemographic and reproductive factors. In contrast, in a country such as Nigeria, the highest maternal mortality was observed in the 26-30-year age group, with 29.9% in the 26-30-year age group and 24.7% in the 31-35-year age group [11]. Our findings reinforce the need for targeted antenatal and intrapartum care for older mothers and those with limited education or obstetric risk factors.

Nearly all women in our sample sought care, yet the vast majority of deaths still occurred in health facilities. This pattern suggests that quality and timeliness of care are important correlates of maternal outcomes. A modelling study using data from 81 LMICs estimated that if high‑quality health systems could deliver a core set of evidence‑based maternal and newborn interventions to those already seeking care, maternal deaths would decline by about 28% [12]. Similarly, the multi‑country registry study mentioned above found that hospital delivery and physician attendance were associated with higher mortality risk after adjustment [12], likely reflecting referral of high‑risk cases to facilities that were poorly resourced to manage complications. These findings align with qualitative evidence that women often reach multiple facilities yet still die in contexts where shortages of equipment, blood products, and trained staff are reported [13]. Improving the readiness and quality of facility‑based care, including the rapid recognition of obstetric emergencies, availability of life‑saving supplies, and skilled personnel, may be more strongly associated with improved outcomes than simply increasing facility birth rates.

The three delays framework proved helpful in interpreting our results. The long decision‑to‑seek‑care interval (mean=3.81 days) may be associated with low awareness of danger signs, lacking financial means, or being constrained by social norms. Systematic reviews note that poor awareness, economic constraints, cultural beliefs, and lack of family support frequently delay care‑seeking [2]. Geographic barriers were commonly reported in association with the second delay: distance and transportation difficulties were cited as frequent obstacles. The third delay, receiving adequate care, was captured indirectly via referral reasons. Many women were transferred because initial facilities lacked equipment, blood products, or qualified staff. Such resource shortages have been frequently associated with contributors to the third delay [13]. An Ethiopian study of maternal death surveillance reported that the third delay accounted for about 29% of deaths despite initiatives to improve access [14], reinforcing the need for ongoing quality enhancement.

A further nuance in interpreting our findings is the clear difference between delays occurring at home and those within the formal healthcare system. Our argument for the comparatively short delays among home deaths is that they likely reflect common community practices in Pakistan, where families first attempt familiar home remedies and where local providers, often living within the same area, can reach patients quickly. By contrast, the longer delays observed among facility deaths point to the fact that families usually seek hospital care only once the condition has become severe and then encounter additional system-level barriers such as travel time, triage queues, and limited obstetric readiness. Studies from similar low-resource settings have reported this same pattern, noting that swift community response can coexist with substantial facility-based delays driven by shortages of staff, equipment, or blood products [13]. We observe that while generalizability is difficult for these points and the delays are context-specific, the focus should still be on how to address these issues and tailor them to the specific challenges of each region. A study from Kenya serves as a good example where a "health navigator" intervention significantly reduced delays in recognizing the need for care and reaching care in remote maternal and neonatal emergencies [15].

These insights have clear policy implications. Community education campaigns can help women and families recognize obstetric danger signs and encourage timely decision‑making. Transport vouchers, maternity waiting homes, and ambulance services can mitigate geographic barriers. Crucially, investments are needed to improve the readiness of health facilities ensuring blood banks, emergency obstetric equipment, and adequately trained staff is available to reduce third delays. Some scholars argue that the traditional three delays model should be supplemented with a focus on continuity of care across facilities and over time [16].

This study's strengths include the use of nationally representative data and detailed verbal autopsy records, allowing a comprehensive analysis of demographic, temporal, and contextual factors. However, limitations must be acknowledged. The secondary dataset contained missing values, resulting in different sample sizes across analyses. The verbal autopsy instrument does not record the exact time from facility arrival to treatment; thus, we used referral reasons to a secondary facility as an indirect measure for the third delay. Misclassification of causes of death may have occurred, particularly for indirect or incidental causes. Because the study design was cross‑sectional, causal relationships cannot be established. Future prospective studies could validate these findings and evaluate interventions targeting specific delays.

## Conclusions

Maternal mortality in Pakistan reflects a complex interplay of socioeconomic inequities, systemic deficiencies, and obstetric complications. While reaching a health facility offers some protection, the high proportion of facility-based deaths underscores the need to improve the quality and continuity of care. Interventions that simultaneously address delayed decision‑making, transport barriers, and facility readiness alongside sustained investment in maternal health services may be important components associated with reductions in preventable maternal deaths and advancing health equity in Pakistan and similar low‑resource settings. This study provides valuable insights that can guide the development of targeted interventions aimed at addressing the multifaceted drivers of maternal mortality, ultimately contributing to the advancement of maternal health equity and universal access to quality maternal healthcare services.
